# Dr. Younger and Implantation of Teeth

**Published:** 1887-01

**Authors:** C. A. Marvin


### DR. YOUNGER AND IMPLANTATION OF TEETH.

BY C. A. MARVIN, D. D. S.

Having been favored with an invitation to witness the operation of implantation by Dr. Younger on two occasions recently, I have been asked to give my impressions. The teeth inserted were a right upper first bicuspid, and left lateral incisor, for a young lady, and a right upper lateral incisor for one of our own profession. The bicuspid was planted about two weeks before the other two, at Dr. Jarvie's office; the two incisors, November 4th, at the office of Dr. Hill. The *modus operandi* having already been described in this journal, it is not necessary to consume time and space in repeating it. As to the theory of Dr. Younger, I have only this to say: It is too late in this day of scientific revelations to exclaim, "It is false," for we are constantly confronted with scientific facts which cannot be accounted for except upon the very theories claimed by those who present the facts, and which are seen to be accountable when the theory is acknowledged. This being so, it is not the part of wisdom to deny a theory which leads directly to the result seen, unless another, equally direct and more reasonable, can be substituted. It will not do to say, "I do not believe it," for the cases are before us, and "facts are stubborn things."

There is an alternative theory which I have been inclined to adopt, viz., that the implanted tooth is cemented in its newly-formed socket by the osseous exudation from the freshly cut bone. But this theory is rudely shaken by the fact that the gum borders are found to be firmly connected with the neck of the newly inserted tooth, which condition of things would not necessarily follow the mere cementing of the root. No, there must be vital action to produce this result.

As I understand it, the theory is that the pericementum covers the root and is firmly attached to it. The outer surface of this membrane retains its characteristics as when freshly torn from its former attachments. It may be dry, withered, so shrunken as to be almost imperceptible, and still possess those characteristics. Now, if it can be rehabilitated, put again in a receptive condition, and then placed where the surroundings are favorable for renewed nourishment, and have that nourishment supplied from a vital source, itself vital, warm, appropriate, a renewal of vital action may be expected, and firmness of attachment, living attachment, ensue.

It goes without saying that all this is contrary to the long-accepted laws of physiological action. We have all been instructed differently, and many of us are exceedingly tenacious of the views thus acquired. But here we are brought face to face with a different theory; operations are boldly and confidently performed before our eyes; cases are presented for our examination and we are asked to observe critically, to test carefully, to think seriously, before we accept. Indeed, we are not solicited to accept at all. Dr. Younger is no suppliant. He simply comes before the profession and says: "Gentlemen, I am persuaded that the implantation of natural teeth in artificial sockets in the human jaw can successfully be performed." With no undue boastfulness of manner, but with perfect frankness, he shows just how he does it, explains his theory, gives his reasons, conceals nothing, claims nothing of personal excellence, charges nothing. His manner challenges our admiration. His manipulation is direct, dexterous, confident. His generosity in giving to the profession the results of years of study, speaks the liberality of the man.

The test of time has yet to be passed. Meanwhile, it is only just to furnish to every case every accessory favorable to its success. In our examinations of cases that we are allowed to see, we should be mindful of this fact, and by no rough handling defeat or hinder the healing process of kindly nature. We ought to wish the operation and its inventor success, not failure. Hence the delicate touch, the gentle handling, the careful avoidance of harshness, when examining a newly implanted tooth, should prove the sincerity and worthiness of our wish. The small boy who pulled up his beans every day to see if they had sprouted, deserved ridicule no more than the dentist, who seizes and violently wrenches a newly implanted tooth to see if it has grown fast, deserves censure.

And it is just here, if anywhere, that I feel inclined to criticise Dr. Younger a little. He is not mindful enough of conditions and accessories. He does not exercise care enough in selecting the teeth to be implanted, but consents to perform the operation with as much readiness when the tooth to be inserted is not an appropriate one, as when all things are favorable. He may have perfect confidence in his success—he doubtless has—but to onlookers it seems hardly wise to ignore any condition that would contribute to a good result. Especially in the infancy of a line of practice, when its efficiency is yet to be established, is this so. Dr. Younger's good nature runs away with his judgment. He does not like to disappoint those who have come together to see him operate. While this is very kind in him, it does him an injustice.

These operations are a new departure in dental surgery, novel, unique, wonderful, and we shall eagerly watch for the result after years have gone by and the time-test is fully satisfied. Meanwhile, it is safe to say, the highest and best sentiment in the profession inclines toward Dr. Younger in warm sympathy, and utters itself in a sincere wish that the operation introduced by him may prove an undoubted success, and be adopted as a legitimate department of the dental surgery of the future.

